# Prognostic value of preoperative hydronephrosis in patients with bladder cancer undergoing radical cystectomy: A meta-analysis

**DOI:** 10.1371/journal.pone.0222223

**Published:** 2019-09-12

**Authors:** Zhaowei Zhu, Jia Zhao, Yinghui Li, Chen Pang, Zhanwei Zhu, Xuepei Zhang

**Affiliations:** 1 Department of Urology, The First Affiliated Hospital of Zhengzhou University, Zhengzhou, Henan, PR China; 2 Center for Geriatrics, Yidu Central Hospital of Weifang, Qingzhou, Shandong, PR China; 3 Department of Urology, People’s Hospital of Gongyi City, Zhengzhou, Henan, PR China; 4 Department of Urology, Nanshi Hospital of Nanyang, Nanyang, Henan, PR China; 5 Department of Urology, People’s Hospital of Huaxian, Huaxian, Henan, PR China; London School of Hygiene and Tropical Medicine, UNITED KINGDOM

## Abstract

**Background:**

Hydronephrosis is a common finding in patients with bladder cancer. The aim of the study was to appraise the prognostic value of preoperative hydronephrosis in bladder cancer patients undergoing radical cystectomy.

**Methods:**

We conducted a literature search using PubMed and Embase databases in Aug 2018. Summary hazard ratios (HRs) with 95% confidence intervals (CIs) were calculated using fixed-effect or random-effects models. The primary endpoint was overall survival (OS). Secondary endpoints were cancer-specific survival (CSS) and recurrence-free survival (RFS).

**Results:**

Overall, 13 studies published between 2008 and 2018 including 4,820 patients were selected for the meta-analysis. The age of bladder cancer patients ranged from 27 to 90.4 years, and the overall proportion of males was 72.5%. Preoperative hydronephrosis was reported in 27.4% of patients. The pooled HR was statistically significant for OS (HR, 1.36; 95% CI [1.20–1.55]) and CSS (HR, 1.64; 95% CI [1.33–2.02]), with no heterogeneity among the enrolled studies. Patients with bilateral hydronephrosis showed a poorer CSS compared to those with no hydronephrosis (HR 5.43, 95% CI [3.14–9.40]). However, there was no difference in CSS between no hydronephrosis and unilateral hydronephrosis groups (HR 1.35, 95% CI [0.84–2.14]). Despite a tendency towards poorer RFS (HR, 1.27; 95% CI [0.96–1.96]), the results demonstrated no significant association between presence of preoperative hydronephrosis and RFS after radical cystectomy.

**Conclusion:**

This meta-analysis indicates that preoperative hydronephrosis is significantly associated with poorer OS and CSS after radical cystectomy for patients with bladder cancer. Preoperative hydronephrosis has a stronger effect on CSS in patients with bilateral hydronephrosis. The presence of preoperative hydronephrosis not only predicts prognosis, but may also help to identify patients who benefit the most from neoadjuvant chemotherapy.

## Introduction

Bladder cancer is one of the most common urological cancers worldwide [1]. Radical cystectomy (RC) represents the standard treatment in patients with muscle-invasive or high-risk non-muscle-invasive bladder cancer. Despite undergoing RC, up to 50% bladder cancer patients still experience cancer recurrence and death after RC [2]. Platinum-based neoadjuvant chemotherapy before RC has been regarded as evidence-based treatment to improve prognosis in bladder cancer patients [3,4]. However, it is very difficult for doctors to determine eligible patients due to the poor prognostic value of the traditional TNM staging system. Thus, easily accessible clinical features which are related to survival after RC may facilitate patient counseling and clinical decision making.

Bartsch found that preoperative hydronephrosis in bladder cancer was an independent prognostic factor for recurrence-free survival (RFS) [5]. Recently, many studies have provided conflicting results to the topic [6–18]. Yet the literature is ambiguous as some researches have shown that preoperative hydronephrosis is not related to survival of bladder cancer patients [11–13,15,17]. Thus, we performed a meta-analysis to appraise the prognostic significance of preoperative hydronephrosis after RC for bladder cancer.

## Materials and methods

### Search strategy

We carried out a literature search using the PubMed and Embase databases in Aug 2018. The following keywords were used: “bladder cancer”, “urothelial cancer”, “radical cystectomy”, “preoperative”, “hydronephrosis”, “prognostic value”, “survival” and “prognosis”. This literature search was limited to human studies without limitation of publication year. References of related review papers were examined to identify additional eligible researches. The present meta-analysis was performed according to the Preferred Reporting Items for Systematic Reviews and Meta-Analyses statement guidelines [19] (S1 Table).

### Study selection

Two investigators independently performed study selection (Z.J. and L.Y.H.). Disagreements were settled by another author (Z.Z.W). We used titles and abstracts to select researches which met the initial study inclusion criteria. Full-text articles were utilized when titles and abstracts were not sufficient to ascertain whether the research fulfilled the inclusion criteria.

In the study, we only enrolled researches which met the following criteria: (1) must present information about the preoperative hydronephrosis; (2) must explore the relation between preoperative hydronephrosis and the survival of patients with bladder cancer; (3) must provide risk estimates, such as hazard ratio (HR), with 95% confidence intervals (CIs). Commentaries, editorials, and conference proceedings which did not undergo peer review were also excluded. Finally, we enrolled 13 studies in our meta-analysis. We appraised the study quality using the following items: assessment of hydronephrosis, outcome evaluation, follow-up time, loss to follow-up, and number of adjustment factors [20]. In the present study, the maximum quality score was 10 points, and researches with quality score ≥ 5 points were regarded as high quality [20].

### Data extraction

For each enrolled research, the detailed information was collected: the first author’s last name, study design, study location, recruitment period, population size, percent of hydronephrosis, age (years), gender (male/female), follow-up (month), outcome, and risk estimates with 95% CIs.

### Statistical methods

We used HRs and 95% CIs to assess the association between preoperative hydronephrosis and overall survival (OS), cancer-specific survival (CSS), and RFS for patients with bladder cancer. We used the Cochran Q test and I^2^ statistics to evaluate heterogeneity among researches [20,21]. For the Q statistic, a p value of less than 0.10 was used as an indication of the presence of heterogeneity; for I^2^, a value > 50% was considered a measure of severe heterogeneity [20,21]. Summary HRs with 95% CIs were calculated using fixed-effect or random-effects models [22]. The decision for choosing fixed-effect or random-effects models must be based on clinical knowledge and the aim of the study.

Publication bias was evaluated using a funnel plot of a trial’s effect size against the SE. Because funnel plots have several limitations and represent only an informal approach to detect publication bias, we further carried out formal testing using the test proposed by the Begg’s adjusted rank correlation test and by the Egger’s regression test [20,21,23,24]. All statistical analyses were performed using STATA, version 11.0 (STATA, College Station, TX, USA). A two-tailed p value of less than 0.05 was considered to be statistically significant [20,21].

## Results

### Study selection

**Fig 1:**
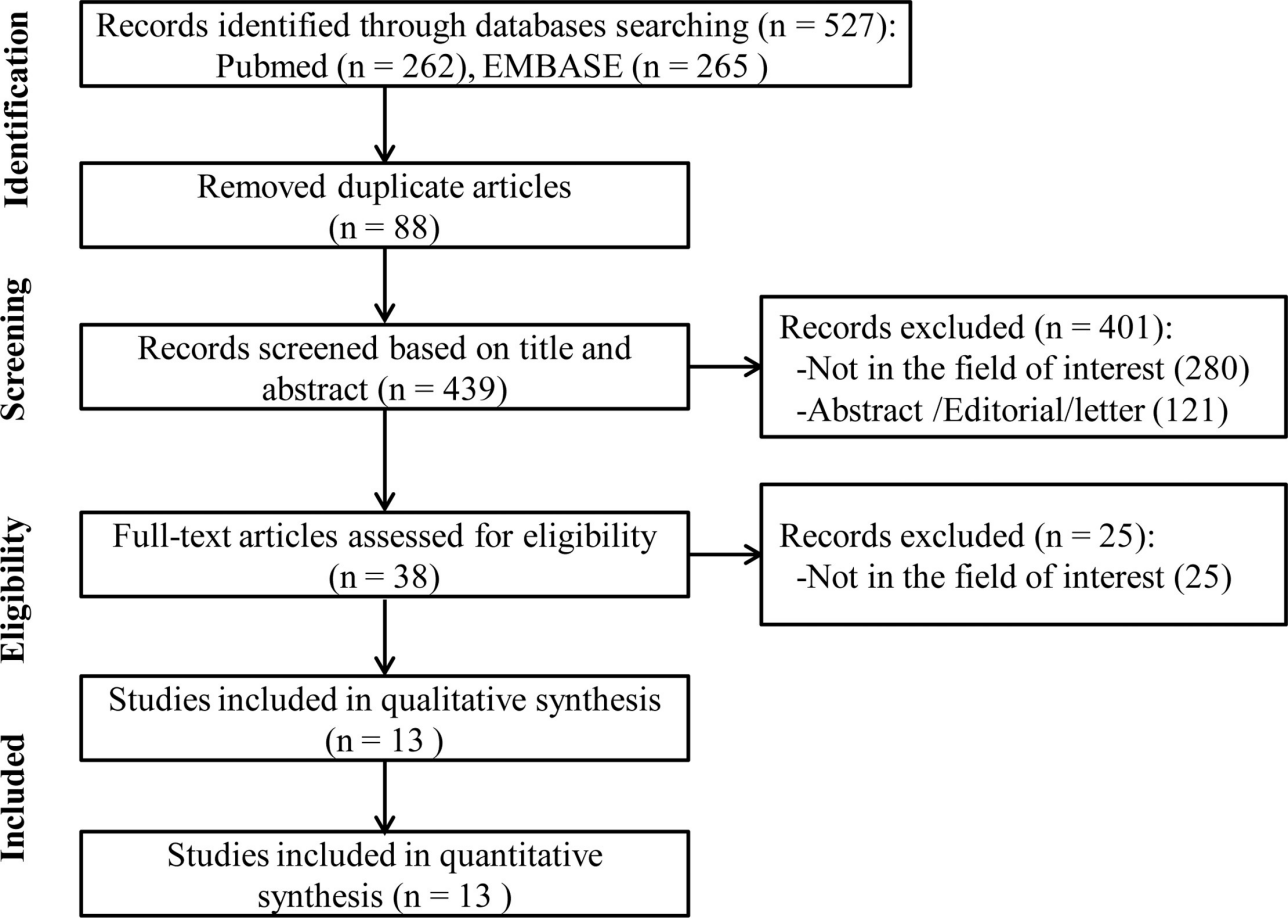
Flow diagram for study selection process.

We identified 527 unique references through literature search. After checking the full text of 38 articles, 13 studies fulfilled the inclusion criteria and were enrolled in the present meta-analysis [6–18]. All the studies had a retrospective design, except one study which consisted of both prospective and retrospective part [11]. Four of the researches were carried out in North America, four in Asia, three in Europe, and one in19 centers across Europe, Canada and the USA. Fig 1 depicts the selection process of eligible studies in the meta-analysis.

### Study population

The main characteristics of the 13 enrolled researches were shown in Table 1. Overall, there were 4820 patients included in the 13 studies which evaluated the influence of preoperative hydronephrosis on mortality of bladder cancer patients after RC. The patient age ranged from 27 to 90.4 years, and the proportion of men was 72.5%. Of the 13 studies, 10 directly provided HR and 95% CI values [6,8,9,11–14,16–18]; one paper provided RR value [7], and one paper provided OR value [10], which were used to estimate HR (S2 Table). In addition, one article reported estimated effects and p values, but do not give CIs [15]. Thus, we used the method of Altman DG et al. [25] to obtain the CIs.

**Table 1:** Baseline characteristics of the included studies.

Study	Design	Country	Recruitment period	Sample size	Hydronephrosis (%)	Age, y	Gender (m/f)	Follow-up, mo	Outcome
**Canter**	Retrospective	US	1988–2003	306	24.2	Md: 65.3 (Range: 35–84)	238/68	Md: 45.6 (Range: 0–223)	OS/DSS
**Chapman**	Retrospective	US	1996–2006	308	34	Mn: 66.4 (Range: 29.7–90.4)	236/72	NA	OS
**Resorlu**	Retrospective	Turkey	1990–2007	241	21.6	Mn: 59.8 (Range: 29–83)	214/27	Mn: 34 (Range: 1–175)	CSS
**Kim**	Retrospective	Korea	1986–2005	406	20.9	Md: 60.8 (Range: 27–79)	360/46	Md: 66.3 (Range: 3–232)	CSS
**Stimson**	Retrospective	US	2001–2007	753	32	Md: 69 (IQR 15)	584/169	NA	OS
**Asadauskiene**	Prospective Retrospective	Lithuania	2000–2008	46	52.2	Md: 60.5 (95% CI: 57.8–64.1)	43/3	NA	OS
**Hofner**	Retrospective	Germany	1990–2009	328	23	Md: 64 (Range: 40–87)	230/98	Md: 8.7 (Range: NA)	CSS
**Lin**	Retrospective	China	2003–2010	126	31	Md: 60 (Range: 32–85)	110/16	Md: 23 (Range: 2–89)	RFS
**Gondo**	Retrospective	Japan	2000–2009	189	20.1	Mn: 68.4 (Range: 38–85)	158/31	Mn: 34.4 (Range: 2.1–127.9)	DSS
**Mitra**	Retrospective	US	1971–2009	414	23.2	NA	0/414	Md: 12.2 (Range: 1.0–27.8)	RFS/OS
**Hirasawa**	Retrospective	Japan	2003–2015	136	22.1	Mn: 68.6 (Range: NA)	112/24	Mn: 46.7 (Range: NA)	CSS
**Soria**	Retrospective	NA	1988–2003	354	27	Md: 66.3 (IQR: 60.3–71.9)	287/67	Md: 123 (IQR: 79–180)	RFS/OS/CSS
**Vasdev**	Retrospective	Europe, Canada and US	2000–2013	1213	30.3	Md: 64 (IQR: 54–71)	921/292	Md: 19.2 (Range: 6–42)	OS

CSS, cancer-specific survival; DSS, disease-specific survival; f, female; IQR: interquartile range; m, male; Md, median; Mn, mean; OS, overall survival.

### Survival outcomes

**Fig 2:**
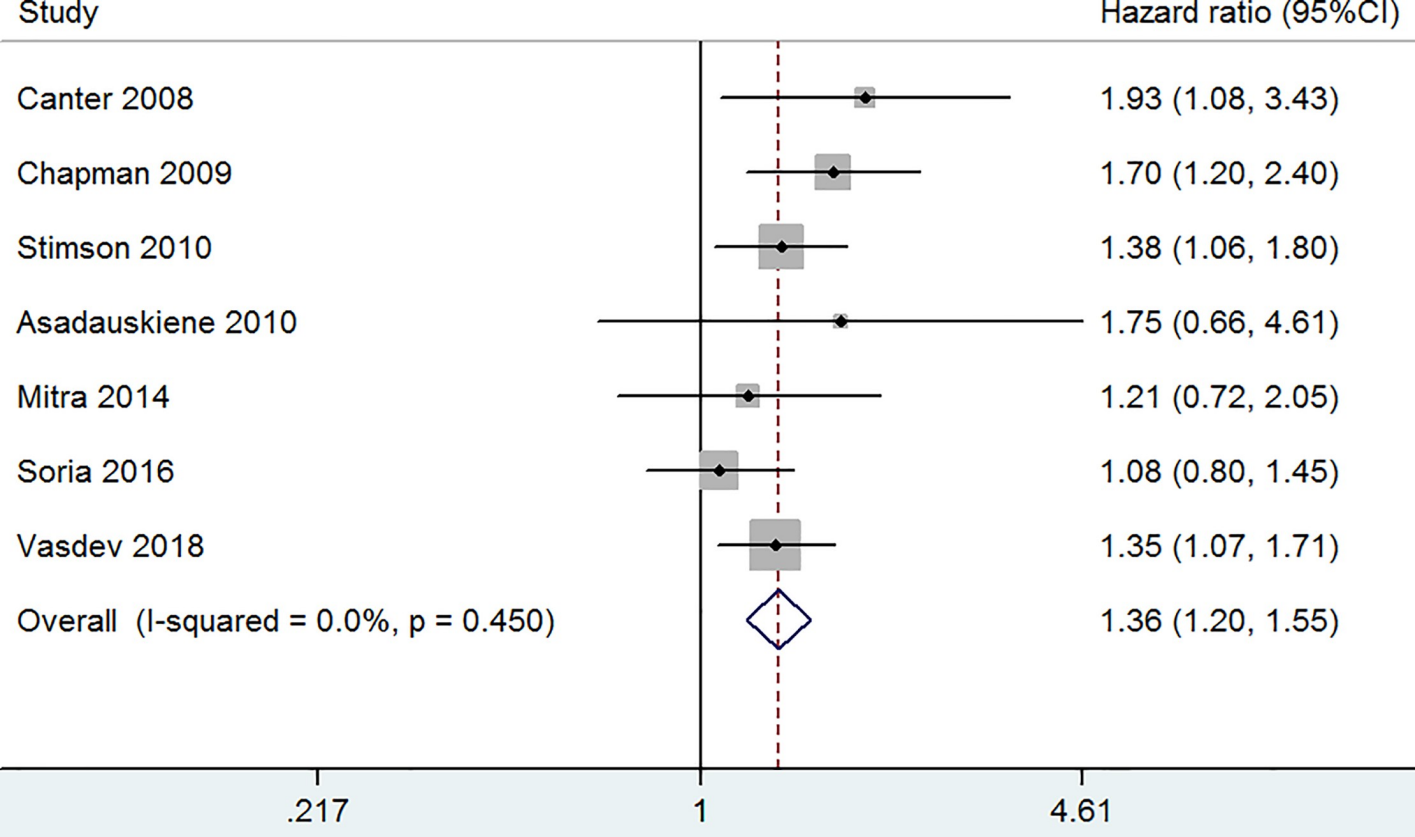
The hazard ratio (HR) of preoperative hydronephrosis associated with OS in bladder cancer patients.

Of the seven studies [6,7,10,11,15,17,18] reporting the relationship between preoperative hydronephrosis and OS after RC, the combined HR and 95% CI for bladder cancer patients after RC was 1.36 (95% CI [1.20–1.55], p ≤ 0.001) with no heterogeneity (I^2^ = 0.0%, p = 0.450) (Fig 2, fixed-effect model).

**Fig 3:**
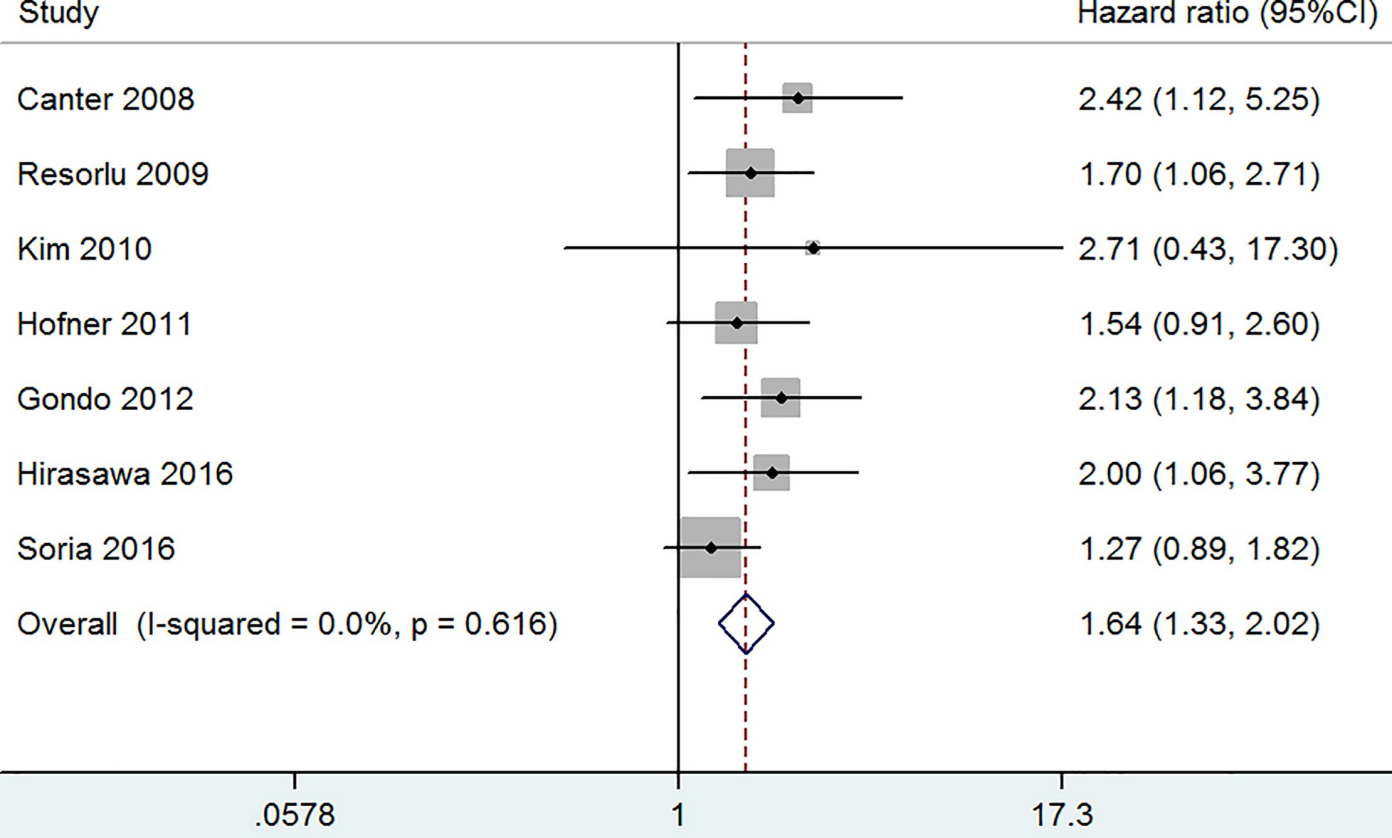
The hazard ratio (HR) of preoperative hydronephrosis associated with CCS in bladder cancer patients.

The combined results of the seven studies [6,8,9,12,14,16,17] were analyzed to investigate the overall association between preoperative hydronephrosis and CSS after RC. As shown in Fig 3, the combined HR and 95% CI for CSS provided in the seven studies was 1.64 (95% CI [1.33–2.02], p ≤ 0.001) with no heterogeneity among the seven studies (I^2^ = 0.0%, p = 0.616) (Fig 3, fixed-effect model).

**Fig 4:**
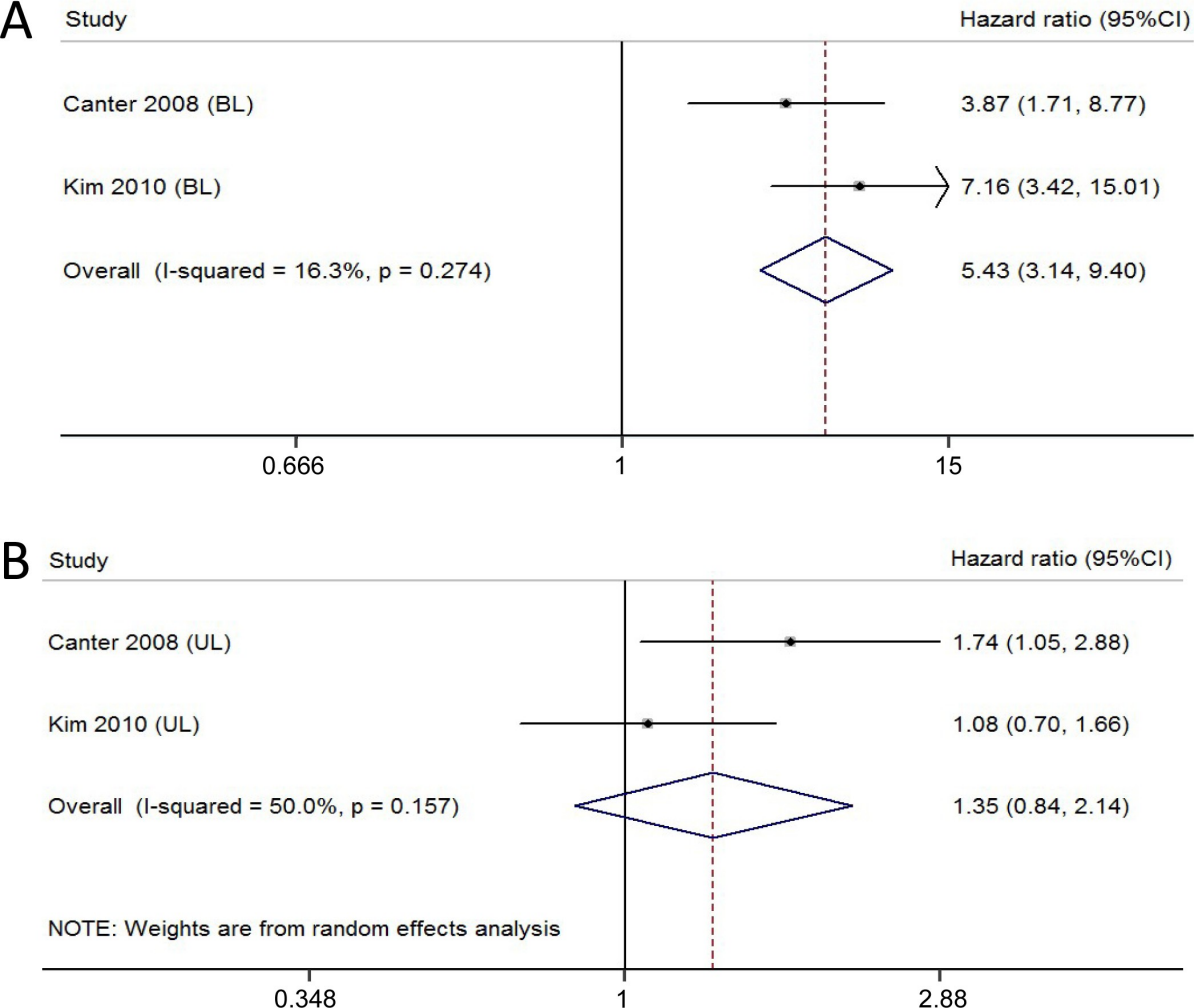
Forest plots showing the association between bilateral or unilateral hydronephrosis and CSS in bladder cancer patients. (A) Bilateral hydronephrosis (BL); (B) Unilateral hydronephrosis (UL).

Bladder cancer patients with bilateral hydronephrosis displayed a poorer CSS compared to those with no hydronephrosis (HR 5.43, 95% CI [3.14–9.40], p ≤ 0.001), with slight heterogeneity among the two studies (I^2^ = 16.3%, p = 0.274) (Fig 4A, fixed-effect model). However, no significant difference was found in CSS between no hydronephrosis and unilateral hydronephrosis groups (HR 1.35, 95% CI [0.84–2.14], p = 0.212), with moderate heterogeneity among the two studies (I^2^ = 50.0%, p = 0.157) (Fig 4B, random-effects model).

**Fig 5:**
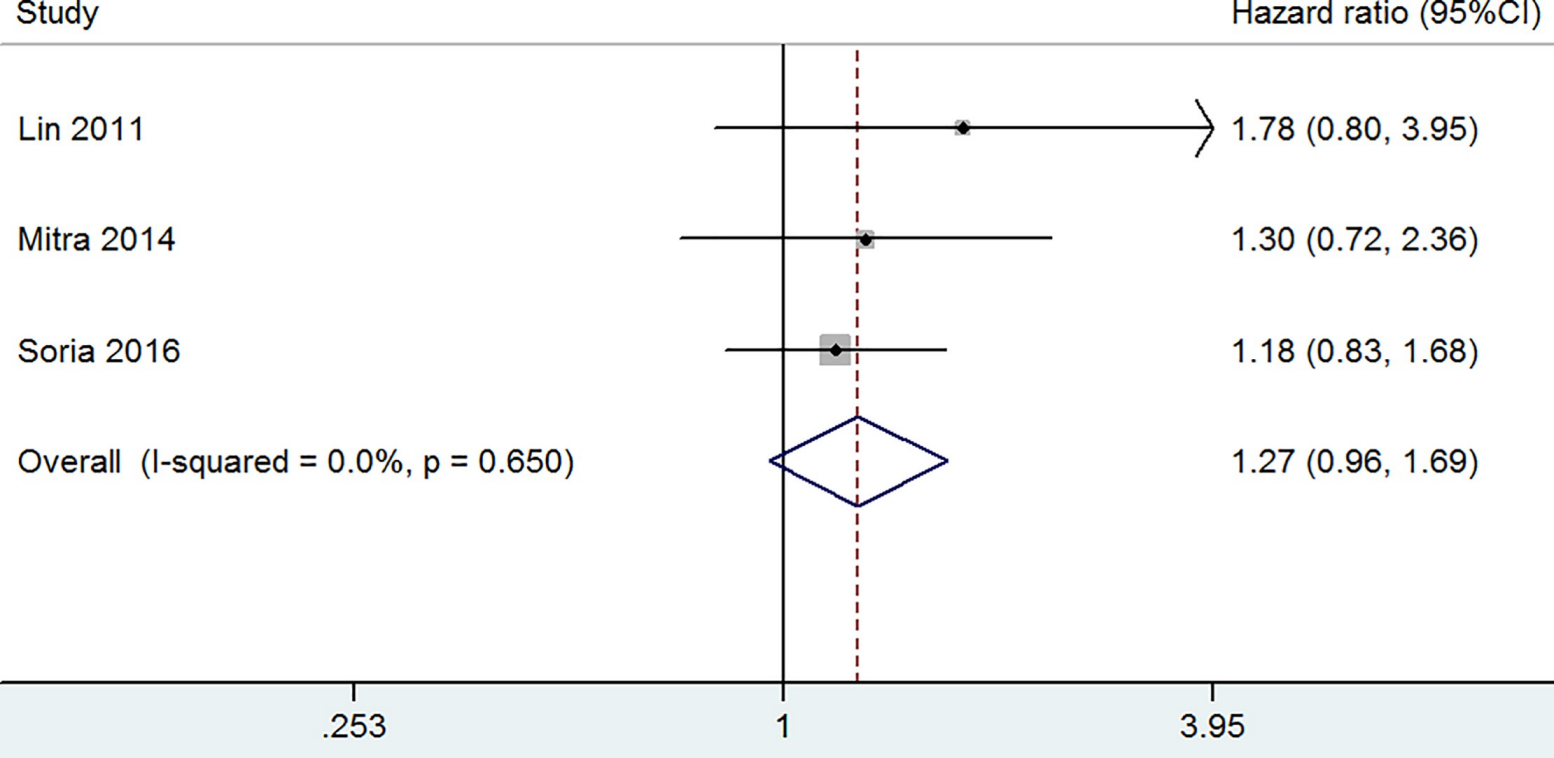
The hazard ratio (HR) of preoperative hydronephrosis associated with RFS in bladder cancer patients.

The combined HR and 95% CI for RFS in the three studies [13,15,17] was 1.27 (95% CI [0.96–1.96], p = 0.098) with no heterogeneity (I^2^ = 0.0%, p = 0.650) (Fig 5, fixed-effect model). Despite a tendency towards poorer RFS, there was no significant association between preoperative hydronephrosis and RFS after RC.

### Publication bias

**Fig 6:**
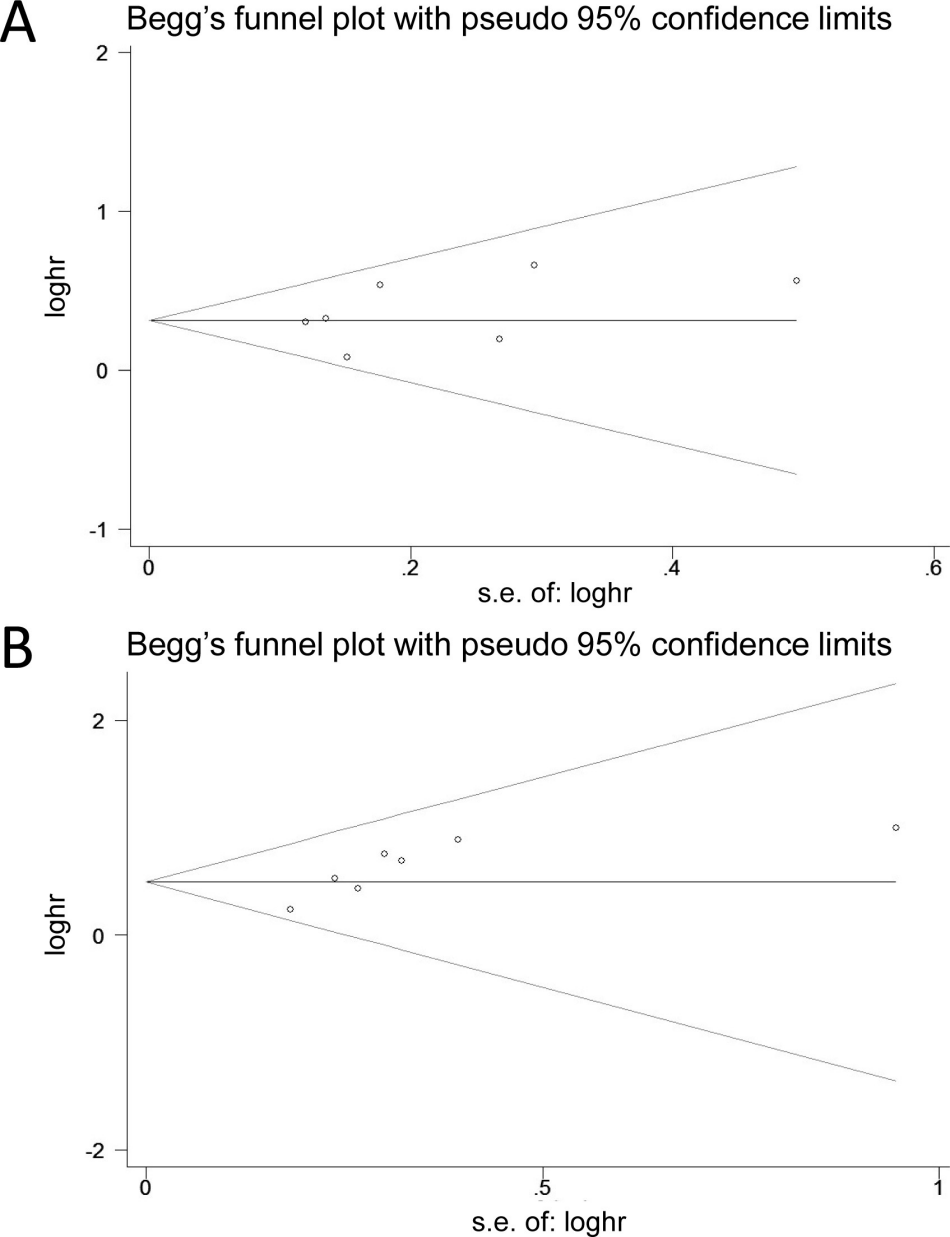
Funnel plots were used to evaluate publication bias on OS and CSS. Begg’s test and Egger’s test were not significant indicating that no significant bias was observed. (A) OS; (B) CSS.

We only assessed the publication bias for OS and CSS using Begg’s test and Egger’s test (Fig 6A and 6B). No funnel plot asymmetry was observed for the relationship between preoperative hydronephrosis and OS and CSS after RC. P values for Begg’s test was 0.548 and Egger’s test was 0.397 with respect to OS, suggesting that our analyses were stable for OS. Begg’s test (p = 0.133) and Egger’s test (p = 0.041) for CSS also suggested a low possibility of publication bias.

## Discussion

The representative characteristic of bladder carcinoma includes a high recurrence rate, distinctive morbidity and mortality. Seisen and colleagues assessed significant predictors of intravesical recurrence after radical nephrouretectomy from a systematic review of the literature and meta-analysis and identified patient-, tumor- and treatment-specific characteristics which should be systematically assessed to guide postoperative decision-making [26]. Identification of clinical and pathological features of bladder cancer mortality risk is also crucial for selecting optimal treatment. Numerous researches have explored potential prognostic predictors for bladder cancer patients so as to guide treatment decisions and improve survival after RC. It has been demonstrated that tumor stage and lymphovascular invasion are the main prognostic factors in bladder cancer [16,27].

Previous researches have reported various results relating preoperative hydronephrosis to upper tract urothelial carcinoma [28]. Tian et al. conducted a comprehensive meta-analysis of nineteen relevant studies and found that preoperative hydronephrosis was associated with increased risk and poor survival in patients with upper tract urothelial carcinoma [28]. Accumulating studies have suggested that preoperative hydronephrosis are also common in bladder cancer patients. Bladder cancer might cause ureteral orifice obstruction which leads to hydronephrosis. Thus, the tumor stage might be associated with the degree of hydronephrosis. Noteworthy, hydronephrosis has been found in association with a high probability of advanced tumors [5]. Although various researches have been carried out to investigate the prognostic significance of preoperative hydronephrosis, the evidence are still equivocal and conflictive for bladder cancer. Kim et al. reported that preoperative hydronephrosis was associated with higher tumor stage and lymphatic metastasis [9]. Stimson also showed that hydronephrosis was an independent predictor of advanced bladder cancer stage [10].

To the best of our knowledge, this is the first meta-analysis to systematically assess the association between preoperative hydronephrosis and survival of bladder cancer patients. The research aggregated outcomes of 4820 patients with bladder cancer undergoing RC in 13 individual studies. Thus, this meta-analysis has higher statistical power than each study. Noteworthy, there was no heterogeneity among the studies in Fig 2, Fig 3 and Fig 5. We just observed slight heterogeneity in Fig 4A and moderate heterogeneity in Fig 4B. Based on clinical knowledge and the goal of the investigation, we used fixed-effect models to calculate summary HRs with 95% CIs except for Fig 4B. Considering that only two studies were included in Fig 4, it was impossible to perform meta-regression or sub-group analyses.

We observed that preoperative hydronephrosis was present in 27.4% of the patients. Combined analysis of the enrolled researches indicated that preoperative hydronephrosis is a significant predictor for poor OS and CSS in bladder cancer patients. The meta-analysis estimates represent the true HR for the association of preoperative hydronephrosis with survival outcomes after radical cystectomy. Thus, it would be reasonable to administer neoadjuvant chemotherapy before surgery for bladder cancer patients with preoperative hydronephrosis.

RFS duration was generally calculated from date of RC to date of first clinical recurrence or last follow-up [29]. Although hydronephrosis significantly affected the RFS for all the patients on the log-rank test, the multivariate analysis showed that hydronephrosis was not an independent prognostic factor for RFS except for pathological stage and lymph node status [13]. Mitra and colleagues also stated that hydronephrosis was unrelated to higher risk of recurrence in multivariable Cox proportional hazards models [15]. Similarly, preoperative hydronephrosis was not significantly related to RFS on multivariable analysis [17]. Accordingly, when the three studies were included in a meta-analysis, we observed that preoperative hydronephrosis did not predict worse RFS, but there was a trend that it might reach statistical significance with a larger sample size.

Canter et al. demonstrated that the presence of bilateral hydronephrosis conferred an approximately three fold risk of patients experiencing a decrease in CSS [6]. The HR for unilateral hydronephrosis also manifested prognostic information, albeit to a lesser degree [6]. In another study, however, no significant difference was found in CSS between unilateral hydronephrosis and no hydronephrosis groups [9]. Our results from a meta-analysis also suggest unilateral hydronephrosis was unrelated to poor CSS in patients with bladder cancer after RC. However, only two studies were enrolled in the meta-analysis for assessing the prognostic value of unilateral hydronephrosis. Further researches are required to fully appraise the prognostic significance of unilateral and bilateral hydronephrosis for patients with bladder cancer.

Our study has some limitations that warrant consideration when interpreting these results. First, most of the enrolled researches were retrospective in nature. However, prospective randomized controlled trials which investigate the prognostic value of preoperative hydronephrosis are not available. Second, the use of published aggregate data compared with individual patient data meta-analysis limits the ability to perform meaningful analysis of subgroup effects [30]. Third, confounding cannot be fully excluded as a potential explanation for the observed association, because our analyses were based on observational studies [21]. Finally, inherent in any meta-analysis of published data is the possibility of publication bias, that is small studies with null results tend not to be published [21]. However, the results obtained from funnel plot analysis and formal statistical tests did not provide evidence for such bias [21].

## Conclusions

This meta-analysis indicates that preoperative hydronephrosis is significantly associated with poorer OS and CSS after RC for patients with bladder cancer. Preoperative hydronephrosis has a stronger effect on CSS in patients with bilateral hydronephrosis. However, no significant association was found between preoperative hydronephrosis and RFS after RC. The presence of preoperative hydronephrosis not only predicts prognosis, but may also help to identify patients who benefit the most from neoadjuvant chemotherapy. Further prospective studies are required to provide a precise prognostic significance of preoperative hydronephrosis in bladder cancer.

## Supporting information

S1 Table PRISMA 2009 checklist. (DOC)

S2 Table Raw data. (XLSX)
